# The Wonders of Your Watch

**Published:** 1892-08

**Authors:** 


### THE WONDERS OF YOUR WATCH.

Open your watch and look at the little wheels, springs and screws, each an indispensable part of the whole wonderful machine. Notice the busy little balance wheel as it flies to and fro unceasingly, day and night, year in and year out. This wonderful little machine is the result of hundreds of years of study and experiment. The watch carried by the average man is composed of 98 pieces, and its manufacture embraces more than 2,000 distinct and separate operations. Some of the smallest screws are so minute that the unaided eye cannot distinguish them from steel filings or specks of dirt. Under a powerful magnifying glass a perfect screw is revealed. The slit in the head is 2–1000 of an inch wide. It takes 308,000 of these screws to weigh a pound, and a pound is worth $1,585. The hair-spring is a strip of the finest steel, about 9½ inches long and 1–100 inch wide and 27–10,000 inch thick. It is coiled up in spiral form and finely tempered.

The process of tempering these springs was long held as a secret by the few fortunate ones possessing it, and even now is not generally known. Their manufacture requires great skill and care. The strip is gauged to 20–1000 of an inch, but no measuring instrument has as yet been devised capable of fine enough gauging to determine beforehand by the size of the strip what the strength of the finished spring will be. A 1–20,000 part of an inch difference in the thickness of the strip makes a difference in the running of a watch of about six minutes per hour.

The value of these springs, when finished and placed in watches, is enormous in proportion to the material from which they are made. A comparison will give a good idea. A ton of steel made up into hair-springs when in watches is worth more than twelve and one-half times the value of the same weight of pure gold. Hair-spring wire weighs one-twentieth of a grain to the inch. One mile of wire weighs less than half a pound. The balance gives five vibrations every second, 300 every minute, 18,000 every hour, 432,000 every day, and 157,680,000 every year. At each vibration it rotates about one and one-fourth times, which makes 197,100,000 revolutions every year. In order that we may better understand the stupendous amount of labor performed by these tiny workers let us make a few comparisons. Take, for illustration, a locomotive with six-foot driving wheels. Let its wheels be run until they have given the same number of revolutions that a watch does in one year, and they will have covered a distance equal to twenty-eight complete circuits of the earth. All this a watch does without other attention that winding once every twenty-four hours.

